# Structure of the first representative of Pfam family PF04016 (DUF364) reveals enolase and Rossmann-like folds that combine to form a unique active site with a possible role in heavy-metal chelation

**DOI:** 10.1107/S1744309110007517

**Published:** 2010-07-06

**Authors:** Mitchell D. Miller, L. Aravind, Constantina Bakolitsa, Christopher L. Rife, Dennis Carlton, Polat Abdubek, Tamara Astakhova, Herbert L. Axelrod, Hsiu-Ju Chiu, Thomas Clayton, Marc C. Deller, Lian Duan, Julie Feuerhelm, Joanna C. Grant, Gye Won Han, Lukasz Jaroszewski, Kevin K. Jin, Heath E. Klock, Mark W. Knuth, Piotr Kozbial, S. Sri Krishna, Abhinav Kumar, David Marciano, Daniel McMullan, Andrew T. Morse, Edward Nigoghossian, Linda Okach, Ron Reyes, Henry van den Bedem, Dana Weekes, Qingping Xu, Keith O. Hodgson, John Wooley, Marc-André Elsliger, Ashley M. Deacon, Adam Godzik, Scott A. Lesley, Ian A. Wilson

**Affiliations:** a Joint Center for Structural Genomics, http://www.jcsg.org, USA; b Stanford Synchrotron Radiation Lightsource, SLAC National Accelerator Laboratory, Menlo Park, CA, USA; c National Institutes of Health, Bethesda, MD, USA; d Program on Bioinformatics and Systems Biology, Burnham Institute for Medical Research, La Jolla, CA, USA; e Department of Molecular Biology, The Scripps Research Institute, La Jolla, CA, USA; f Protein Sciences Department, Genomics Institute of the Novartis Research Foundation, San Diego, CA, USA; g Center for Research in Biological Systems, University of California, San Diego, La Jolla, CA, USA; h Photon Science, SLAC National Accelerator Laboratory, Menlo Park, CA, USA

**Keywords:** structural genomics, domains of unknown function, rare metals, siderophores, pterins

## Abstract

The crystal structure of Dhaf4260 from *Desulfitobacterium hafniense* DCB-2 was determined by single-wavelength anomalous diffraction (SAD) to a resolution of 2.01 Å using the semi-automated high-throughput pipeline of the Joint Center for Structural Genomics (JCSG) as part of the NIGMS Protein Structure Initiative (PSI). This protein structure is the first representative of the PF04016 (DUF364) Pfam family and reveals a novel combination of two well known domains (an enolase N-terminal-like fold followed by a Rossmann-like domain). Structural and bioinformatic analyses reveal partial similarities to Rossmann-like methyltransferases, with residues from the enolase-like fold combining to form a unique active site that is likely to be involved in the condensation or hydrolysis of molecules implicated in the synthesis of flavins, pterins or other siderophores. The genome context of Dhaf4260 and homologs additionally supports a role in heavy-metal chelation.

## Abstract 2

The crystal structure of the first representative of DUF364 family reveals a combination of enolase N-terminal-like and C-terminal Rossmann-like folds. Analysis of the interdomain cleft combined with sequence and genome context conservation among homologs, suggests a unique catalytic site likely involved in the synthesis of a flavin or pterin derivative.

## 1. Introduction

To extend the structural coverage of proteins for which the biological function is unknown and cannot be deduced by homology (*i.e.* domains of unknown function; DUFs), targets were selected from the Pfam (Finn *et al.*, 2008 ▶) protein family PF04016 (DUF364). DUF364 homologs are encountered in proteobacteria, firmicutes, actinobacteria, cyanobacteria, thermotogae and a number of archaea. Here, we report the crystal structure of Dhaf4260, the first structural representative of this family, which was determined using the semiautomated high-throughput pipeline of the Joint Center for Structural Genomics (JCSG; http://www.jcsg.org; Lesley *et al.*, 2002 ▶) as part of the NIGMS Protein Structure Initiative (PSI). The *Dhaf4260* gene of *Desulfitobacterium hafniense* DCB-2 encodes a protein with a molecular weight of 27.7 kDa (residues 1–251) and a calculated iso­electric point of 5.6. *Desulfitobacterium* spp. are anaerobic bacteria that are capable of dehalogenating organic compounds and have been studied for their potential in bioremediation processes (Villemur *et al.*, 2006 ▶; El Fantroussi *et al.*, 1998 ▶).

## 2. Materials and methods

### 2.1. Protein production and crystallization

Clones were generated using the Polymerase Incomplete Primer Extension (PIPE) cloning method (Klock *et al.*, 2008 ▶). The gene encoding Dhaf4260 (UniProt B8FUJ5, see Supplementary Material^1^) was amplified by polymerase chain reaction (PCR) from *D. hafniense* DCB-2 genomic DNA using *PfuTurbo* DNA polymerase (Stratagene) and I-PIPE (Insert) primers (forward primer, 5′-ctgtacttccag­ggcATGTGGGAGATCTATGACGCCATGATC-3′; reverse primer, 5′-aattaagtcgcgttaTTTTTTTATGGTCACCTTCTGTCCCGCG-3′; target sequence in upper case) that included sequences for the predicted 5′ and 3′ ends. The expression vector pSpeedET, which encodes an amino-terminal tobacco etch virus (TEV) protease-cleavable expression and purification tag (MGSDKIHHHHHH­ENLYFQ/G), was PCR-amplified with V-PIPE (Vector) primers (forward primer, 5′-taacgcgacttaattaactcgtttaaacggtctccagc-3′; reverse primer, 5′-gccctggaagtacaggttttcgtgatgatgatgatgatg-3′). V-PIPE and I-­PIPE PCR products were mixed to anneal the amplified DNA fragments together. *Escherichia coli* GeneHogs (Invitrogen) com­petent cells were transformed with the V-PIPE/I-PIPE mixture and dispensed onto selective LB-agar plates. The cloning junctions were confirmed by DNA sequencing. Expression was performed in a selenomethionine-containing medium at 310 K with suppression of normal methionine synthesis. At the end of fermentation, lysozyme was added to the culture to a final concentration of 250 µg ml^−1^ and the cells were harvested and frozen. After one freeze–thaw cycle, the cells were sonicated in lysis buffer [50 m*M* HEPES pH 8.0, 50 m*M* NaCl, 10 m*M* imidazole, 1 m*M* tris(2-carboxyethyl)phosphine–HCl (TCEP)] and the lysate was clarified by centrifugation at 32 500*g* for 30 min. The soluble fraction was passed over nickel-chelating resin (GE Healthcare) pre-equilibrated with lysis buffer, the resin was washed with wash buffer [50 m*M* HEPES pH 8.0, 300 m*M* NaCl, 40 m*M* imidazole, 10%(*v*/*v*) glycerol, 1 m*M* TCEP] and the protein was eluted with elution buffer [20 m*M* HEPES pH 8.0, 300 m*M* imidazole, 10%(*v*/*v*) glycerol, 1 m*M* TCEP]. Since prior testing had revealed that the designed protease site in the expression and purification tag did not cleave with TEV protease, protease was not added to the protein preparation. The eluate was buffer-exchanged with crystallization buffer (20 m*M* HEPES pH 8.0, 200 m*M* NaCl, 40 m*M* imidazole, 1 m*M* TCEP) using a PD-10 column (GE Healthcare) and concentrated to 5 mg ml^−1^ by centrifugal ultrafiltration (Millipore). Dhaf4260 was crystallized at 277 K by mixing 200 nl protein solution with 200 nl crystallization solution and equilibrating against 50 µl reservoir volume using the nanodroplet vapor-diffusion method (Santarsiero *et al.*, 2002 ▶) with standard JCSG crystallization protocols (Lesley *et al.*, 2002 ▶). The crystallization reagent consisted of 1.0 *M* LiCl and 0.1 *M* citrate pH 5.0. Ethylene glycol (1,2-ethanediol) was added to the crystal as a cryoprotectant to a final concentration of 20%(*v*/*v*). A diamond-shaped crystal of approximate dimensions 100 × 100 × 100 µm was harvested at room temperature after 46 d at 277 K and cryocooled in liquid nitrogen. Initial screening for diffraction was carried out using the Stanford Automated Mounting system (SAM; http://smb.slac.stanford.edu/facilities/hardware/SAM/UserInfo; Cohen *et al.*, 2002 ▶) at the Stanford Synchrotron Radiation Lightsource (SSRL, Menlo Park, California, USA). The data were indexed in the hexagonal space group *P*6_1_.

The oligomeric state of Dhaf4260 in solution was determined using a 1 × 30 cm Superdex 200 column (GE Healthcare) coupled with miniDAWN static light-scattering (SEC/SLS) and Optilab differential refractive-index detectors (Wyatt Technology). The mobile phase consisted of 20 m*M* Tris pH 8.0, 150 m*M* sodium chloride and 0.02%(*w*/*v*) sodium azide.

### 2.2. Data collection, structure solution and refinement

Single-wavelength anomalous diffraction (SAD) data were collected on beamline BL9-2 at the SSRL at a wavelength corresponding to the peak of a selenium SAD experiment. The data set was collected at 100 K with a MAR 325 CCD detector using the *Blu-Ice* data-collection environment (McPhillips *et al.*, 2002 ▶). The SAD data were integrated and reduced using *XDS* and scaled and merged with the program *XSCALE* (Kabsch, 1993 ▶). Initial substructure solution was performed with *SHELX* (Sheldrick, 2008 ▶) and phases were refined with *SOLVE* (Terwilliger & Berendzen, 1999 ▶), with a mean figure of merit of 0.24 (0.37 to 2.9 Å) for ten selenium sites. Density modification and automated model building were performed with *RESOLVE* (Terwilliger, 2003 ▶) and produced a trace for 443 residues (82%) with 424 side chains built and sequence-assigned. Model completion and refinement were performed with *Coot* (Emsley & Cowtan, 2004 ▶) and *REFMAC* 5.2 (Winn *et al.*, 2003 ▶). Refinement included experimental phase restraints in the form of Hendrickson–Lattman coefficients from *SOLVE*, loose NCS restraints (positional weights 5.0 and thermal weights 10.0) and TLS refinement with one TLS group per chain. Data-reduction and refinement statistics are summarized in Table 1 ▶. 

### 2.3. Validation and deposition

**Figure 1:**
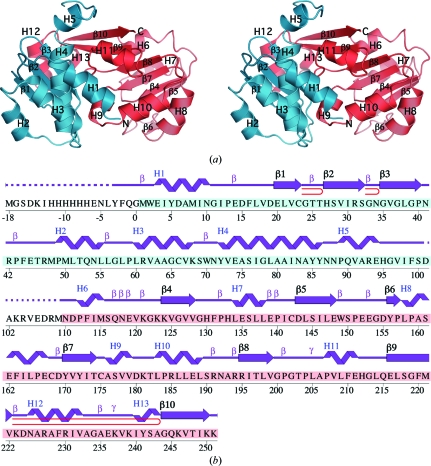
Crystal structure of Dhaf4260 from *D. hafniense*. (*a*) Stereo ribbon diagram of the Dhaf4260 monomer. The N-terminal domain is colored cyan and the C-terminal domain is colored salmon. Helices H1–H13 and β-strands β1–β10 are indicated. (*b*) Diagram showing the secondary-structure elements of Dhaf4260 superimposed on its primary sequence. The designation of secondary-structure elements is in accord with *PDBsum* (http://www.ebi.ac.uk/pdbsum). For Dhaf4260, helices are labeled sequentially (H1, H2, H3 *etc*.) with α-helices H1–H6, H8–H10 and H12 and 3_10_-helices H7, H11 and H13, β-strands are numbered sequentially (strands β1–β3 form the first sheet and strands β4–β10 form the second sheet), β-turns are labeled β, γ-turns are labeled γ and β-hairpins are indicated as red loops. The unmodeled sequence, which is disordered in the electron-density map, is indicated by a dashed line. Residues from the N-terminal domain are highlighted in cyan and residues from the C-terminal domain are in salmon.

Analysis of the stereochemical quality of the model was accomplished using *AutoDepInputTool* (Yang *et al.*, 2004 ▶), *MolProbity* (Davis *et al.*, 2007 ▶), *SFCHECK* v.4.0 (Collaborative Computational Project, Number 4, 1994 ▶) and *WHAT IF* v.5.0 (Vriend, 1990 ▶). Protein quaternary-structure analysis was performed using the *PISA* server (Krissinel & Henrick, 2007 ▶). Fig. 1 ▶(*b*) was adapted from an analysis using *PDBsum* (Laskowski *et al.*, 2005 ▶) and all other figures were prepared with *PyMOL* (DeLano Scientific). Atomic coordinates and experimental structure factors for Dhaf4260 at 2.01 Å have been deposited in the PDB and are accessible under code 3l5o.

## 3. Results and discussion

### 3.1. Overall structure

The crystal structure of Dhaf4260 (Fig. 1 ▶ *a*) was determined to 2.01 Å resolution using the single-wavelength anomalous dispersion (SAD) method. Data-collection, model and refinement statistics are summarized in Table 1 ▶. The final model includes two Dhaf4260 protomers [491 residues; molecule *A* contains residues 1–102 and 110–251 in addition to three residues from the N-terminal expression and purification tag (residues −2*A* to 0*A*), and molecule *B* contains residues 1–102 and 115–251 and five residues from the N-terminal expression and purification tag (residues −4*B* to 0*B*)], six ethylene glycol molecules, four imidazole molecules, two chloride ions and 239 water molecules in the asymmetric unit. The electron density was insufficient to model the loop connecting the N- and C-terminal domains (residues 103–109 in molecule *A* and residues 103–114 in molecule *B*) and the remainder of the N-terminal expression and purification tags (residues −18 to −3 in molecule *A* and −18 to −5 in molecule *B*). Side-chain atoms from Phe(−2), Phe44, Glu45, Thr46, Arg47, Gln53, Gln90, Asp101, Glu135, Leu137, Arg194, Lys223, Lys237 and Lys239 in chain *A* and Leu(−4), Tyr(−3), Gln(−1), Ser100, Asp101, SeMet115, Ser116, Gln117, Asn118, Lys121, Lys123, Lys137, Glu153, Lys237, Lys239 and Lys250 in chain *B* were omitted owing to weak electron density. The Matthews coefficient (*V*_M_; Matthews, 1968 ▶) was 2.6 Å^3^ Da^−1^ and the estimated solvent content was 53.2%. The Ramachandran plot produced by *MolProbity* (Davis *et al.*, 2007 ▶) showed that 97.5% of the residues were in favored regions and 99.8% were in allowed regions. The single outlier, Gln117 from chain *B*, was located in a region of poor electron density.

Dhaf4260 is a two-domain α+β protein (Fig. 1 ▶). SCOP describes the N-terminal domain (residues 1–102) as adopting an enolase N-­terminal domain-like fold (http://scop.mrc-lmb.cam.ac.uk/scop/data/scop.b.e.bca.A.A.html) characterized by three helices (H1–H3) with up–down–up topology and a three-stranded antiparallel β-sheet (β1–β3). The C-terminal domain (residues 110–251) adopts a Rossmann-like fold that is described in SCOP as PLP-dependent transferase-like (http://scop.mrc-lmb.cam.ac.uk/scop/data/scop.b.d.jg.html). The typical NAD(P)-binding Rossmann fold is characterized by a three-layer α/β/α sandwich structure with a parallel sheet and a 321456 topology, but does not contain the additional antiparallel strand observed in Dhaf4260 (strand order 3214567). The PLP-dependent transferase-like fold is characterized by a similar sandwich that contains a seven-stranded mixed β-sheet (β4–β10 in Dhaf4260) with the seventh strand (β10 in Dhaf4260) antiparallel to the rest. However, there is only partial congruence between the sheet topology of the PLP-dependent transferase-like fold (strand order 3245671) and that observed in Dhaf4260. Further, the lysine to which the co-factor is linked in the PLP-dependent transferase-like fold is absent in Dhaf4260-like proteins.

**Figure 2:**
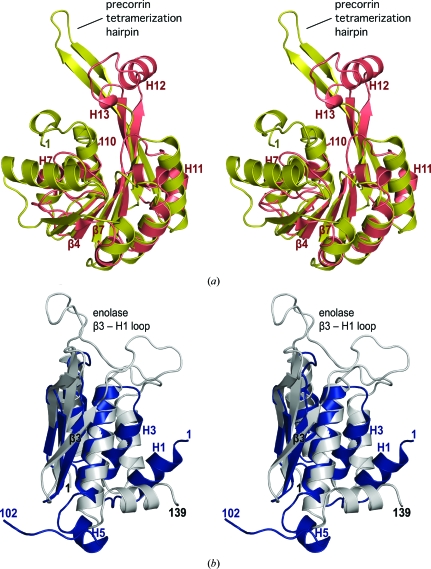
Stereo ribbon diagram showing the structural superposition of (*a*) the C-terminal domain of Dhaf4260 (PDB code 3l5o; residues 110–251; salmon) and precorrin-8w methyltransferase from *Methanobacterium thermoautotrophicum* (MT0146; PDB code 1f38; residues 1–186;  gold) and (*b*) the N-terminal domain of Dhaf4260 (PDB code 3l5o; residues 1–102; blue) and the enolase N-terminal domain from *Saccharomyces cerevisiae* (PDB code 4enl; residues 1–139; gray). The precorrin methyltransferase and enolase regions implicated in oligomerization and substrate binding are indicated.

A search of intact Dhaf4260 with *FATCAT* (Ye & Godzik, 2004 ▶) indicates that the strongest structural similarity is to precorrin-8w methyltransferases [PDB codes 1f38 (Keller *et al.*, 2002 ▶) and 2yxd (B. Padmanabhan, Y. Bessho & S. Yokoyama, unpublished work)], with C^α^ r.m.s.d.s of 3.1 and 3.2 Å over 170 and 173 residues, respectively (sequence identity of 9%) for these Rossmann-like methyltransferases involved in the anaerobic pathway of cobalamin (vitamin B_12_) biosynthesis (Scott & Roessner, 2002 ▶; Keller *et al.*, 2002 ▶). The similarity maps to the C-terminal domain of Dhaf4260 and involves both fold and topology, with the exception of the last two strands, which are inverted (the strand order is 3214567 for Dhaf4260 and 3214576 for the precorrin methyltransferases). Other differences include an extra helix between precorrin methyltransferase strands β2 and β3 (equivalent to Dhaf4260 strands β5 and β6), the addition of Dhaf4260 helices H12 and H13, which are replaced by a long hairpin that is involved in tetramerization and ligand binding in the precorrin methyltransferases (Keller *et al.*, 2002 ▶), and an additional Dhaf4260 helix H11 in the loop between strands β8 and β9 (strands 5 and 6 in the precorrin methyltransferases; Fig. 2 ▶ *a*). In addition, a similar mode of tetramerization is not possible in Dhaf4260 because the corresponding interface is involved in interactions with the N-terminal domain.

The N-terminal domain of Dhaf4260 shows strong structural similarity to the N-terminal domain of enolase (PDB code 4enl; Lebioda *et al.*, 1989 ▶), with a C^α^ r.m.s.d. of 1.9 Å over 60 residues, but a sequence identity of only 8%. Structural differences involve an extra N-terminal helix (H1) in Dhaf4260 and different orientations of helices H3 and H5 (Fig. 2 ▶ *b*). A weak similarity of this domain to several RNA-binding proteins was also observed, including ribosomal protein L22 (PDB code 1bxe; Unge *et al.*, 1998 ▶; C^α^ r.m.s.d. of 3.1 Å over 61 residues with 10% sequence identity) and a double-stranded RNA-specific editase (PDB code 1di2; Ryter & Schultz, 1998 ▶; C^α^ r.m.s.d. of 2.1 Å over 101 residues and a sequence identity of 7%). Although in both of these latter cases the β-sheet and long central helix (H4) systematically superimpose well, along with one or two of the outer helices (H2, H3), the connectivity is different, limiting the scope for functional inference.

In bacteria, the enolase N-terminal-like fold is found in a number of epimerases and racemases that catalyze stereochemical inversion in biological molecules. The enolase superfamily, which comprises mandelate racemase (MR), muconate-lactonizing enzyme (MLE) and enolases, is a group of functionally related enzymes each of which is organized into two domains: a substrate-specificity-determining capping N-terminal domain followed by a TIM barrel that contains the metal-ion ligands and acid/base catalysts at the C-­terminal ends of the β-strands (Gerlt & Babbitt, 2001 ▶). The long β3–H1 loop that connects the third strand to the first helix closes onto the active site upon substrate binding (Fig. 2 ▶ *b*). The corresponding loop in Dhaf4260 (β3–H2) is much shorter. Many enolases are dimers and the dimerization interface is conserved among prokaryotes and eukaryotes, where dimerization is proposed to play a role in promoting subunit stability (Kühnel & Luisi, 2001 ▶). Some of the residues involved in dimerization are from the N-terminal domain. For example, residues from the first two strands of the enolase N-­terminal domain β-sheet and residues preceding the H1 helix interact with residues from the enolase C-terminal domain in the adjacent protomer. In Dhaf4260 such an oligomerization mode is not possible because the C-terminal domain is not the same.

Size-exclusion chromatography of Dhaf4260 in combination with static light scattering indicates a mixture of oligomerization states, with a tetramer being the predominant quaternary form. However, crystal-packing analysis of the Dhaf4260 structure only supports a monomer or dimer and did not identify any higher order oligomeric state in this crystal form. This discrepancy between the oligomerization state in solution and in the crystal could arise from the crystallization selecting monomeric or dimeric states from the observed mixture of states in solution, or the crystallization conditions could alter the distribution of states observed. The presence of the 19-residue N-terminal expression and purification tag might also alter the oligomerization state relative to the wild-type protein. Thus, these results are inconclusive as to the true nature of the biologically relevant oligomeric state of this protein.

### 3.2. A unique catalytic site

**Figure 3:**
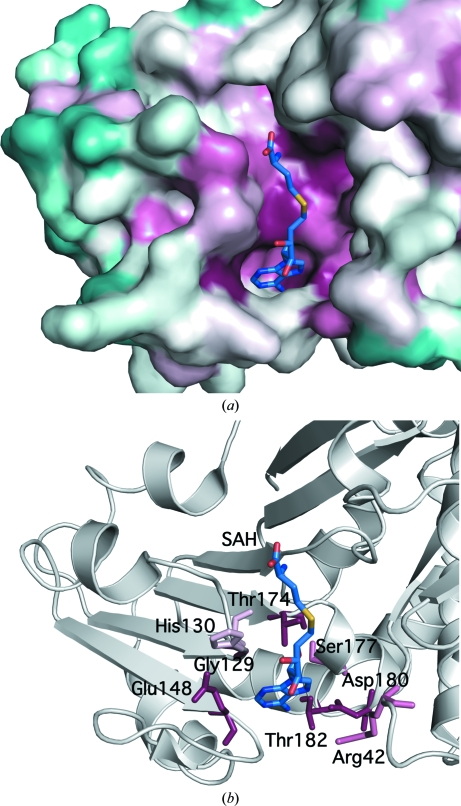
The interdomain pocket forms a unique catalytic site. (*a*) Surface representation of the Dhaf4260 domain interface colored by sequence conservation according to *ConSurf* (Landau *et al.*, 2005 ▶). High conservation among DUF364 homologs is indicated in maroon and low conservation is indicated in turquoise. A docked *S*-­adenosyl-l-homocysteine (SAH) molecule is shown in ball-and-stick representation. Docking was based on its superposition with MT0146 (PDB code 1l3i; Keller *et al.*, 2002 ▶). (*b*) Ribbon representation of Dhaf4260 in the same orientation as in (*a*). Highly conserved Dhaf4260 residues are shown in ball-and-stick representation and are labeled.

A search of the N-terminal domain of Dhaf4260 against the Pfam database using the remote protein homology-detection server *HHPred* (Soding *et al.*, 2005 ▶) produced weak hits with a ribosomal RNA methyltransferase family (PF07091; *P*-value 0.0023 over Dhaf4260 residues 3–26, probability 0.10) and a family of RNA polymerase II-associated proteins (PF08620; *P*-value 0.0069 over Dhaf4260 residues 40–88, probability 0.07). The C-terminal domain showed significant homology with PF03446 (*P*-value 9.5 × 10^−5^ over Dhaf4260 residues 123–204, probability 0.91), PF02826 (*P*-value 7.2 × 10^−5^ over residues 119–200, probability 0.81) and PF00670 (*P*-­value 3.3 × 10^−5^ over residues 120–200, probability 0.80). All three families contain NAD-binding domains, with PF00670 being a member of a family of *S*-adenosyl-l-homocysteine (SAH) hydrolases, which are B_12_-dependent enzymes of the activated methyl cycle. Residues that are conserved among all three families and DUF364 are Gly129, Glu148, Thr174 and Asp180 (the numbering is for Dhaf4260). Residues that additionally show high conservation among Dhaf4260 homologs include Gly37, Gly39, Arg42, Asn83, Thr133 and Thr182. Mapping of these residues that are conserved in DUF364 homologs onto the structure of Dhaf4260 shows that they cluster inside a deep pocket (∼660 Å^3^) in the interface between the enolase-like and Rossmann-like domains (Fig. 3 ▶ *a*), suggesting that this region serves as an active site and that DUF364 homologs function as enzymes.

An aspartate or glutamate residue that interacts with the hydroxyl groups of the ribose is the most highly conserved feature of adenosyl (*e.g.* ATP, NAD and *S*-adenosyl-l-methionine) binding sites (Carugo & Argos, 1997 ▶). Asp62 could fulfill this role in MT0146 and is superimposable with Glu148, which is strictly conserved among Dhaf4260 homologs (Fig. 3 ▶ *b*). Other similarities to Rossmann-like folds (Burroughs *et al.*, 2006 ▶) involve the presence of highly conserved polar residues (Thr174, Asp180 and Thr182) in the two orthogonal helices downstream of strand β4 of the Rossmann-like fold (equivalent to strand β7 in Dhaf4260) and a glycine followed by a hydrophobic or aromatic residue (Gly129 and His130) in the classical loop position between strand 1 and helix 1 of the Rossmann-like fold (equivalent to strand β4 and helix H7 in Dhaf4260) (Figs. 1 ▶ and 3 ▶ *b*).

The GGSGG loop that completes the precorrin binding site and is implicated in binding *S*-adenosyl-l-methionine (SAM) through an induced-fit mechanism is absent from Dhaf4260, suggesting a different ligand and a different reaction mechanism. A G*X*G-type signature is observed in a different loop (Gly37, Gly39 and Arg42) bordering one side of the adenine base, with Arg42 (Fig. 3 ▶ *b*) possibly engaged in a similar hydrophobic packing interaction as Arg63 in MT0146. In addition, the Dhaf4260 pocket is both narrower and longer than in Rossmann-like methyltransferases such as MT0146, suggesting that it catalyzes the modification of a longer substrate or the condensation of two molecules.

A chloride ion is present in this cleft in both molecules in the asymmetric unit and is coordinated by the backbone amide of Trp149 and solvent. This chloride-binding site is in a similar location to that of the adenosyl ring of the SAH bound in the MT0146 structure. Since the chloride only makes a single protein contact within the pocket and given the high chloride concentration in the crystallization reagent (1 *M*), this interaction is not likely to be functionally relevant.

A search against a database of nonredundant cognate binding sites using *IsoCleft* (Najmanovich *et al.*, 2008 ▶), a graph-matching algorithm that searches for both geometrical and chemical composition similarities, identified shared features between the Dhaf4260 cavity and the binding sites of proteins implicated in binding vitamin B_12_ (B_12_-dependent glutamate mutase; PDB code 1ccw; 40 atoms in common, Tanimoto similarity score 0.13, *Z* score 3.96, *P*-value 1.11 × 10^−2^; Reitzer *et al.*, 1999 ▶), typical hemes (PDB code 2nap; 37 atoms in common; Tanimoto similarity score 0.160, *Z* score 3.48, *P*-value 2.04 × 10^−2^; Dias *et al.*, 1999 ▶), atypical hemes (PDB code 1q90; 37 atoms in common; Tanimoto similarity score 0.178, *Z* score 3.48, *P*-value 2.04 × 10^−2^; Stroebel *et al.*, 2003 ▶), factor F430 (PDB code 1e6v; 37 atoms in common; Tanimoto similarity score 0.151, *Z* score 3.48, *P*-value 2.04 × 10^−2^; Grabarse *et al.*, 2000 ▶) and both heme and FAD (flavohemoglobin; PDB code 1cqx; 36 atoms in common; Tanimoto similarity score 0.187, *Z* score 3.32, *P*-value 2.50 × 10^−2^; Ermler *et al.*, 1995 ▶). Proteins that bind metals (iron–sulfur clusters, divalent cations) and adenine dinucleosides [bis(adenosine)-5′-pentaphos­phate] and dinucleotides [FAD, NAD(P)] also scored highly.

The corrin ring (four pyrrole subunits) that comprises the core of vitamin B_12_ is chemically similar to the porphyrin found in hemes, but one of the bridging methylene groups is removed. Uroporphyrinogen III is an intermediate in the biosynthesis of vitamin B_12_ and also of heme, siroheme, chlorophylls and factor F430 (Scott & Roessner, 2002 ▶). Hence, all ligands predicted for Dhaf4260 share chemical and structural similarity with flavin or pterin derivatives.

### 3.3. Genome-context analysis

The genome context (http://string.embl.de) of DUF364 homologs shows a high degree of confidence in a predicted functional association with a number of proteins involved in the transport and chelation of rare metals such as iron (WS1133), tungstate (MTH926), vanadium (RPA1384 and RPA1385) and molybdate (MTH924, Mbar_A1307 and amb0153), as well as transcriptional regulators (*e.g.* TetR, LysR, TraR/DksA, CrcB, MerR and PadR) involved in the chemical stress response. Gene neighborhood association with ABC transporters (including both ATPase and membrane-spanning permease subunits) are found with a wide phylogenetic distribution in prokaryotic homologs, suggesting that DUF364 enzymes predominantly act on a soluble substrate, which is likely to be a heavy metal that is transported by these systems. In this context, DUF364 homologs could function in the condensation or hydrolysis of specific side chains in the synthesis of derivatives of flavins, pterins or similar compounds (*e.g.* sidero­phores) that might serve to chelate these metals.

The Dhaf4260 protein family DUF364 (PF04016) contains around 165 homologs that are mostly found in cyanobacteria, actinobacteria, thermotogae and proteobacteria, but are also found in firmicutes and a range of archaea; all of these proteins are approximately 230 residues in length. The availability of further DUF364 member sequences and structures might shed light on the evolutionary history of this intriguing protein family. The information presented here, in com­bination with further biochemical and biophysical studies, should yield valuable insights into the functional role of Dhaf4260. Models for Dhaf4260 homologs can be accessed at http://www1.jcsg.org/cgi-bin/models/get_mor.pl?key=3l5oA.

Additional information about Dhaf4260 is available from *TOPSAN* (Krishna *et al.*, 2010 ▶) at http://www.topsan.org/explore?PDBid=3l5o.

## 4. Conclusions

The first structural representative of the DUF364 family reveals a novel two-domain organization in which an enolase N-terminal-like fold combines with a C-terminal Rossmann-fold-like domain to form a unique catalytic site at the domain interface. Analysis of the genetic context and interdomain cleft suggest a role in heavy-metal uptake, possibly involving the synthesis of a flavin or pterin derivative.

## Supplementary Material

PDB reference: Dhaf4260 from *D. hafniense* DCB-2, 3l5o

Supplementary material file. DOI: 10.1107/S1744309110007517/wd5120sup1.pdf

## Figures and Tables

**Table 1:** Summary of crystal parameters, data-collection and refinement statistics for Dhaf4260 (PDB code 3l5o) Values in parentheses are for the highest resolution shell.

Space group	*P*6_1_
Unit-cell parameters (Å)	*a* = *b* = 120.64, *c* = 75.20
Data collection	
Wavelength (Å)	0.9791 (λ_1_ Se-SAD)
Resolution range (Å)	29.0–2.01 (2.06–2.01)
No. of observations	311344
No. of unique reflections	41624
Completeness (%)	99.9 (99.9)
Mean *I*/σ(*I*)	17.2 (2.5)
*R*_merge_ on *I*† (%)	6.9 (73.0)
*R*_meas_ on *I*‡ (%)	7.3 (81.2)
Model and refinement statistics	
Resolution range (Å)	29.0–2.01
No. of reflections (total)	41583§
No. of reflections (test)	2092
Completeness (%)	100.0
Data set used in refinement	λ_1_
Cutoff criterion	|*F*| > 0
*R*_cryst_¶	0.171
*R*_free_††	0.214
Stereochemical parameters	
Restraints (r.m.s.d. observed)	
Bond angles (°)	1.43
Bond lengths (Å)	0.014
Average isotropic *B* value (Å^2^)	49.4‡‡
ESU§§ based on *R*_free_ (Å)	0.14
Protein residues/atoms	491/3715
Water/other solvent molecules	238/12

† *R*_merge_ = 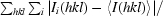 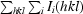.

‡ *R*_meas_ = 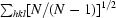 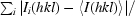 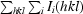 (Diederichs & Karplus, 1997 ▶).

§ The number of unique reflections used in refinement is typically slightly less than the total number that were integrated and scaled. Reflections were excluded owing to systematic absences, negative intensities and rounding errors in the resolution limits and unit-cell parameters.

¶ *R*_cryst_ = 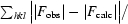 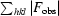, where *F*_calc_ and *F*_obs_ are the calculated and observed structure-factor amplitudes, respectively.

†† *R*_free_ is the same as *R*_cryst_ but for 5.0% of the total reflections that were chosen at random and omitted from refinement.

‡‡ This value represents the total *B* that includes TLS and residual *B* components.

§§ Estimated overall coordinate error (Collaborative Computational Project, Number 4, 1994 ▶; Cruickshank, 1999 ▶).

## Footnotes

1 Supplementary material has been deposited in the IUCr electronic archive (Reference: WD5120).
