# Supplementary material for: Structure of the first representative of Pfam family PF04016 (DUF364) reveals enolase and Rossmann-like folds that combine to form a unique active site with a possible role in heavy-metal chelation
Source: Acta Crystallogr Sect F Struct Biol Cryst Commun. 2010 Jul 6;66(Pt 10):1167–73. doi: 10.1107/S1744309110007517 (PMC2954201; doi:10.1107/S1744309110007517)
Supplement: Supplementary file 1 [file f-66-01167-sup1.pdf]

Supplementary Material

Due to a recent re-assembly of the *Desulfitobacterium hafniense* DCB-2 genome, a number of database reference identifiers including the locus name for the Dhaf4260 protein have changed. Several databases of derived data have not yet been updated and still reference the old identifiers. Supplementary Table S1 is provided to help clarify the relationship between these identifiers. The UniProt Archive database (UniParc) can be useul when resolving obsolete names to existing sequences. In this case, the UniParc id is UPI0000540F56 and can be accessed via the http://www.uniprot.org/uniparc/UPI0000540F56 URL.

Supplementary Table S1. Current and obsolete database and locus identifiers for the Dhaf4260 protein.

UniProtKB	RefSeq	Locus./ ORF Name	EMBL / Genbank	Status	Effective Dates
**B8FUJ5**	**YP_002460701**	**Dhaf_4260**	**ACL22265**	**Current**	**Jan-2009 to present**
Q18YZ7	ZP_01370078	Dhaf_3308	EAT53660	**Obsolete**	Jul-2006 to Jan-2009
Q422F5	ZP_00559375	DhafDRAFT_1477	EAM96800	**Obsolete**	Jul-2005 to Jul-2006
